# Venetoclax-based therapy for relapsed or refractory acute myeloid leukemia: latest updates from the 2022 ASH annual meeting

**DOI:** 10.1186/s40164-023-00424-z

**Published:** 2023-06-30

**Authors:** Xubo Gong, Yi Zhang, Xin He, Milad Moloudizargari, Teng Yu, Lin Wang, Weiwei Liu, Lan Jin, Huiying Xu, Yang Xu, Zhihua Tao, Wenbin Qian

**Affiliations:** 1 Department of Clinical Laboratory, The Second Affiliated Hospital, Zhejiang University School of Medicine, 88 Jiefang Road, Hangzhou, 310000 Zheijang P.R. China; 2 Department of Hematology, The First Affiliated Hospital, Zhejiang University School of Medicine, Hangzhou, 310003 Zheijang P.R. China; 3 Department of Hematological Malignancies Translational Science, Gehr Family Center for Leukemia Research, Hematologic Malignancies and Stem Cell Transplantation Institute, Beckman Research Institute, City of Hope Medical Center, Duarte, CA USA; 4 Department of Hematology, The Second Affiliated Hospital, Zhejiang University School of Medicine, 88 Jiefang Road, Hangzhou, 310009 P.R. China

**Keywords:** Venetoclax, Acute myeloid leukemia, Hypomethylating agents

## Abstract

Venetoclax (VEN), the first selective Bcl-2 inhibitor, has shown efficacy and safety both as monotherapy and in combination with other agents in the treatment of newly diagnosed acute myeloid leukemia (AML), while its role in relapsed or refractory (R/R) disease is not well defined. Here, we reviewed the latest advances of VEN-based therapy for R/R AML from the 2022 American Society of Hematology (ASH) Annual Meeting, including some novel and encouraging regimes, such as VCA, VAH, and HAM regimes, etc. Further research is still needed to fully understand the optimal use of these agents in R/R AML treatment.

**To the editor,**

Relapsed or refractory (R/R) acute myeloid leukemia (AML) is associated with a poor prognosis and limited treatment options [1]. The Bcl-2 inhibitor venetoclax (VEN), in combination with hypomethylating agent (HMA) or low-dose cytarabine, was approved by the FDA in 2020 for newly-diagnosed AML in adults 75 years or older, or in patients with comorbidities precluding intensive chemotherapy [2], while its role in R/R disease was not well defined yet. In this study, we provided an overview of the latest updates of VEN-based therapy for R/R AML from the 2022 American Society of Hematology (ASH) meeting.

## VEN combined with epigenetic drugs for R/R AML

The combination of VEN and HMA in R/R AML were investigated by several studies from different countries, which showed overall response rates (ORR) of 44% [3], 52% [4], and 58.8% [5], respectively. Highlighting the regional difference using this combination for R/R AML. 1-year and 2-year overall survival (OS) was 49.6% (95% CI; 39.9-61.8%) and 39.0% (95% CI; 27.2-55.9%) [5], respectively.

ASTX727 is an oral agent that combines decitabine with the cytidine deaminase inhibitor cedazuridine. The combination of VEN with ASTX727 [6] achieved an ORR of 53%. With a median follow-up of 7 months, the median OS was not reached. It demonstrates that this combination is feasible with significant efficacy in patients with R/R AML.

Chidamide is a subtype-selective benzamide inhibitor of histone deacetylase. In one phase II study [7], the triple combination of VEN, azacitidine (AZA), and chidamide (VCA) achieved an ORR of 75%, and no grade III or IV non-hematologic adverse events (AE) was observed. It shows that VCA is promising with acceptable toxicity for patients with R/R AML.

Homoharringtonine (HHT), a natural alkaloid derived from the cephalotaxus, is a well-defined anti-AML agent. The triplet of VEN, HMA, and HHT (VAH) [8] had significantly higher rate of complete remission (CR)/CR with incomplete count recovery (CRi) (63.8% vs. 40.9%, *P* = 0.003), OS (no reach vs. 16.0 months, *P* = 0.023) and event-free survival (EFS) (7.1 vs. 2.3 months, *P* < 0.001) than VEN + HMA group. Subsequently, the data were updated [9] with a median follow-up of 14.7 months, the median OS was 22.1 months, and EFS was 14.3 months. The 1-year OS was 61.5%, and EFS was 51.0%.

In this series, the novel VCA and VAH regimes appear to have higher response rate, and the efficacy is worth further exploration.

## VEN combined with chemotherapy drugs for R/R AML

Cladribine is an adenosine deaminase resistant analog of adenosine. After one cycle of VEN, cladribine plus low-dose Ara-C (CAV) [10] for R/R AML, the ORR was 90.5%, the estimated 1-year OS was 91.7%, and the EFS was 74.9%. It suggests that the CAV regimen is a very effective salvage strategy in R/R AML.

One phase I/II trial [11] evaluated the combination of VEN with high-dose Ara-C and mitoxantrone (HAM) in patients with R/R AML. The combination was well tolerated and led to CR/CRi of 92%, and 62.5% of evaluable patients were categorized as minimal residual disease (MRD) negative.

Desikan et al. [12] updated results of a phase IIb trial, which evaluated the efficacy of the combination of VEN, fludarabine, Ara-C, G-CSF (FLAG), and idarubicin in R/R AML. It had an ORR of 60% with a composite remission rate (CRc) of 53%. Six-month and 12-month OS was 69% and 60%, respectively. 71% of CRc patients attained a MRD negative remission and 68% of responding patients proceeded to transplant.

In this series, CAV and HAM regimens seem to have deeper remissions. The efficacy and durability of responses need to be further assessed.

## VEN combined with antibodies or targeted drugs for R/R AML

Pivekimab sunirine (PVEK) can target CD123, which is expressed on the majority of AML blasts and leukemic stem cells. In a phase Ib/II study [13], the combination of VEN, AZA, and PVEK achieved an ORR of 51%, with a composite complete remission (CCR) rate of 31%. Compared to patients with prior VEN exposure, VEN-naïve patients had higher ORR (62% vs. 37%) and CCR (47% vs. 11%).

Pegcrisantastase (PegC) is a long-acting crisantastase that exhibits strong anti-leukemia activity through complete consumption of glutamine. An open-label phase Ib clinical trial [14] evaluated the combination of VEN with PegC in R/R AML. The ORR was 64%, with no serious asparaginase-related AE of interest observed. It reveals that VEN-PegC is a well-tolerated regimen.

In conclusion, the 2022 ASH annual meeting presented the new development of VEN-based therapy for R/R AML (Tables 1 and 2), including some novel and encouraging regimes, such as VCA, VAH, and HAM, etc. Further research is still needed to fully understand the optimal use of these agents.

**Table 1:** VEN combined with epigenetic drugs for R/R AML

Authors (reference)	Shahbaz [3]	Mohassel [4]	Weng [5]	Abuasab [6]	Zha [7]	Yu [8]
Regimen	VEN + HMA	VEN + HMA	VEN + HMA	VEN + ASTX727	VEN + AZA + Chidamide	VEN + AZA + HHT
Study type	Pros	Retro	Retro	N/A	Phase II	Pros
NCT number	N/A	N/A	N/A	N/A	N/A	N/A
Study period	N/A	2012–2022	N/A	N/A	N/A	N/A
Age range (years)	20–67	≥ 18	N/A	46–92	35–64	N/A
Patients number	44	25	136	37	29	231
Response rate	ORR 44%, CR/CRi 40%	ORR 52%, CR/CRi 36%	ORR 58.8%, CR/CRi 44.8%	ORR 53%, CR/CRi 46.7%	ORR 75%, CR/CRi 56.3%	CR/CRi 63.8%
Grade ≥ 3 AEs	N/A	Anemia 80%	N/A	N/A	0	N/A
Follow-up (months)	N/A	N/A	N/A	7	N/A	N/A
Survival	N/A	Median OS 9.6 months, median RFS 10.1 months	1- year OS 49.6%, 2-years OS 39.0%	Median OS not reached	N/A	Median OS not reached, median EFS 7.1 months

*AE* adverse events, *AML* acute myeloid leukemia, *AZA* azacitidine, *CR* complete remission, *CRi* CR with incomplete count recovery, *EFS* event-free survival, *HMA* hypomethylating agents, *N/A* not available, *OS* overall survival, *ORR* overall response rate, *Pros* prospective, *R/R* relapsed or refractory, *Retro* retrospective, *RFS* relapse free survival, *VEN* venetoclax

**Table 2:** VEN combined with chemotherapy drugs, antibody drugs or targeted drugs for R/R AML

Authors (reference)	Li [10]	Röllig [11]	Desikan [12]	Daver [13]	Bollino [14]
Regimen	VEN + Cladribine + Ara-C	VEN + HAM	VEN + FLAG-IDA	VEN + AZA + PVEK	VEN + PegC
Study type	N/A	Phase-I/II	Phase IIb	Phase 1b/II	Phase Ib
NCT number	NCT05190549	NCT04330820	N/A	NCT04086264	NCT04666649
Study period	2021–2022	2020–2022	N/A	N/A	N/A
Age range (years)	16–68	40–70	18–68	25–82	24–76
Patients number	21	12	33	71	11
Response rate	ORR 90.5%, CR/CRi 28.6%	CR/CRi 92%	ORR 60%	ORR 51%	ORR 64%
Grade ≥ 3 AEs	N/A	0	N/A	Ferbrile neutropenia 24%	0
Follow-up (months)	N/A	N/A	15.8	N/A	N/A
Survival	Median OS and EFS not reached, estimated 1-year OS 91.7%, estimated 1-year EFS 74.9%	N/A	Median OS 27 months, median DOR not reached. Six-month OS 69%, 12-month OS 60%	N/A	N/A

AE adverse events, *AML* acute myeloid leukemia, *CR* complete remission, *CRi* CR with incomplete count recovery, *EFS* event-free survival, *HAM* high-dose Ara-C and mitoxantrone, *FLAG-IDA* fludarabine, Ara-C, G-CSF and idarubicin, *N/A* not available, *OS* overall survival, *ORR* overall response rate, *PVEK* pivekimab sunirine, *PegC* pegcrisantastase, *R/R* relapsed or refractory, *VEN* venetoclax

## Data Availability

Not applicable.

## Abbreviations

AE: Adverse events
AML: Acute myeloid leukemia
AZA: Azacitidine
CCR: Composite complete remission
CR: Complete remission
CRc: Composite remission rate
CRi: CR with incomplete count recovery
EFS: Event-free survival
FLAG: Fludarabine, Ara-C, and G-CSF
FDA: Food and drug administration
HAM: High-dose Ara-C and mitoxantrone
HHT: Homoharringtonine
HMA: Hypomethylating agents
MRD: Minimal residual disease
ORR: Overall response rate
OS: Overall survival
PegC: Pegcrisantastase
PVEK: Pivekimab sunirine
R/R: Relapsed or refractory
VEN: Venetoclax
